# GAUDIE: Development, validation, and exploration of a naturalistic German AUDItory Emotional database

**DOI:** 10.3758/s13428-023-02135-z

**Published:** 2023-05-23

**Authors:** Katharina Lingelbach, Mathias Vukelić, Jochem W. Rieger

**Affiliations:** 1 Fraunhofer Institute for Industrial Engineering IAO, Nobelstraße 12, 70569 Stuttgart, Germany; 2 Department of Psychology, University of Oldenburg, Oldenburg, Germany

**Keywords:** Affective reactions, Emotion, Arousal, Valence, German auditory stimulus database, Naturalistic stimuli, Emotional speech, Clustering, Validation methods

## Abstract

Since thoroughly validated naturalistic affective German speech stimulus databases are rare, we present here a novel validated database of speech sequences assembled with the purpose of emotion induction. The database comprises 37 audio speech sequences with a total duration of 92 minutes for the induction of positive, neutral, and negative emotion: comedian shows intending to elicit humorous and amusing feelings, weather forecasts, and arguments between couples and relatives from movies or television series. Multiple continuous and discrete ratings are used to validate the database to capture the time course and variabilities of valence and arousal. We analyse and quantify how well the audio sequences fulfil quality criteria of differentiation, salience/strength, and generalizability across participants. Hence, we provide a validated speech database of naturalistic scenarios suitable to investigate emotion processing and its time course with German-speaking participants. Information on using the stimulus database for research purposes can be found at the OSF project repository GAUDIE: https://osf.io/xyr6j/.

Emotional processing, identification, and expression all serve to facilitate interaction by understanding others’ current feelings and intentions. We experience strong emotions, particularly in social scenarios, and attach high relevance and importance to social cues (e.g., Bradley, 2009; Vuilleumier, 2005). Human emotions can be communicated auditorily via sounds like speech. Research pursuing ecological validity has to employ complex, naturalistic stimuli rather than strictly controlled, fragmented, and contextually detached emotional events to investigate how the brain processes stimuli close to everyday emotional situations.

The emotional expression within speech is modulated mainly by two components. One is the prosody, which is formed by the slow modulation of acoustic parameters such as intensity, rate, and pitch (Banse & Scherer 1996; Wilson & Wharton 2006). The other is the lexical content of the utterance. To validly extract the communicated emotion from speech, both components need to be considered. Contradicting information from semantic content and prosody can fundamentally alter the emotion—for instance, in the case of sarcasm or irony (Pell et al., 2011). Ben-David et al. (2016) identified three phenomena when investigating the interaction between prosody and semantic content in emotional speech. First, emotional strength is reinforced for emotionally congruent stimulus presentation (supremacy of congruency). Second, one component cannot be selectively attended and separated from the other (failure of selective attention). Third, prosodic information is weighted as more important than semantic content in emotional speech processing (prosodic dominance).

While non-speech auditory stimuli can be used almost independently of the participants’ native language (Belin et al., 2008; Bradley & Lang, 2007; Yang et al., 2018), emotional speech stimuli, utilizing both prosodic and semantic components, must be language-specific. According to statistics from 2022, approximately 130 million people worldwide have German as their mother tongue or second language. Further, it is the most widely spoken mother tongue in the European Union and even one of the most widely spoken languages worldwide (Statista Research Department, 2022). Although the distribution of German speakers and the active research community in this linguistic area are substantial, there is currently a lack of carefully developed and validated naturalistic stimulus databases for emotion induction. Stimulus material of a naturalistic database should be validated regarding criteria of (i) differentiation of conditions (specificity of the induced emotion), (ii) sufficient salience, intensity, and intrusiveness, and (iii) generalizability across individual differences in order to ensure successful emotional induction (Westermann et al., 1996).

There are several approaches to develop an emotional speech database. Kanske and Kotz (2011) addressed the lexical component by choosing 120 words from the Leipzig Affective Norms for German (LANG) which were validated using the dimensions valence and arousal in written and auditory form. Another common approach is to record semantically neutral sentences or non-comprehendible vocal utterances spoken by (professional) speakers with different emotional expressions, thereby modulating only the component of prosody (Banse & Scherer, 1996; Belin et al., 2008; Burkhardt et al., 2005; Lassalle et al., 2019; Schröder, 2003). Lassalle et al. (2019) who present a comprehensive validated emotional voice database with three subsets (in British English, Swedish, and Hebrew) of uttering sentences, also provide an overview of existing databases for simulated/acted emotional vocal stimuli. Although such recorded stimuli allow a satisfactory recognition of the intended emotion due to distinct acoustic features, they disregard the lexical component. Thus, single words and even contextually detached sentences do not properly approximate the interactive and context-sensitive characteristic of social communication. Consequently, they can be rated as less ecologically valid. In naturalistic speech, the communication of emotion can be more subtle and ambiguous (e.g., by creating contradicting information when voluntarily trying to suppress the emotion in one component due to personal or cultural principles).

Stimuli reflecting naturalistic emotional speech may be created by the following two means. The first is by developing interrogative techniques aiming to engage a person in an emotional conversation, which is a self-related emotional induction. Such self-related stimuli can be considered sufficiently strong since self-attribution might increase the relevance and strength of the emotional cue. However, comparability and generalizability among individuals are then scarcely possible. The second is by presenting already existing engaging speeches addressing the audience or conversations of others to a person, which is experiencing emotions from the listener/observer perspective. The latter approach using the same conversations for each individual facilitates generalizability. However, the perceived emotion by the observer might deviate from the expressed emotion of those persons involved in the conversation. In order to warrant generalizability among individuals and identify the emotion and its strength induced in the observer, it is of utmost importance to validate the emotional speech stimuli using an independent sample. Douglas-Cowie et al. (2003) provide a recent overview of available databases and evaluate them with the following components: (1) scope (i.e., number, gender, and language of speakers, description of the emotions, contextual set-up), (2) naturalness (simulated, scripted, linguistic nature), (3) context (isolated or context-based), (4) descriptors (annotation of the material), and (5) accessibility of the database.

There are several naturalistic speech databases available making use of either movie excerpts with expressive content and broadcasted television programs, or recordings of acted or spontaneously unfolded speeches and conversations (e.g., the HEU Emotion Database; Chen et al., 2021; the Ryerson Audio-Visual Database of Emotional Speech and Song RAVDESS; Livingstone & Russo, 2018; the Russian Acted Multimodal Affective Set RAMAS; Perepelkina et al., 2018; Interactive Emotional Dyadic Motion Capture Database IEMOCAP; Busso et al., 2008; eINTERFACE’05; Martin et al., 2006; Vera am Mittag Database VAM; Grimm et al., 2008). However, most of the databases are developed with the purpose of machine-based emotion recognition (Grimm et al., 2008; Schuller et al., 2009) rather than emotion induction. Consequently, they do not fulfil required criteria of a sufficient level of salience, intensity, and intrusiveness. Thus, despite a great need in the German-speaking area, there is currently no suitable stimulus database available which is particularly developed and validated for emotion induction using naturalistic speech.

Therefore, we aim at providing such a validated naturalistic German speech database, GAUDIE (**G**erman **AUDI**tory **E**motional database), suitable for investigating emotion processing and its time course in naturalistic scenarios. Three types of audio sequences representing speech were selected to induce positive, neutral, and negative emotions. To induce positive emotions, here in the form of humorous and amusing feelings, recordings from various comedy shows were selected. Weather forecasts were selected for the induction of neutral emotion and arguments between couples and relatives from movies or television series were selected to evoke negative emotions.

Different emotion theories and methods can be applied to measure how well an intended emotion is induced by a stimulus. Theories assuming that emotional states are discrete suggest that distinguishable (basic) emotions anger, fear, disgust, sadness, joy, surprise (Ekman, 1992; Izard, 2007), as well as larger selections of emotions (e.g., Plutchik, 2013), may be evoked by stimulus material. This allows us to evaluate stimuli regarding these emotional states using accuracy of categorization as measure. Other theories describe emotions via continuous uni- or multidimensional scales; for instance, valence (i.e., the pleasantness of an event or stimulus), arousal (i.e., the activation evoked), and intensity or dominance (Bakker et al., 2014; Bradley & Lang, 1994; Russell, 1980). There are also attempts to combine the two approaches using continuous intensity ratings per discrete emotion (Plutchik, 2013; Scherer, 2005). Here, we mainly focused on a continuous multidimensional approach using valence, arousal, and dominance to validate the emotion induction, since neuroscientific findings suggest underlying neurophysiological representations of these dimensions, particularly valence and arousal, subserving all (discrete) affective states (Gerber et al., 2008; Greco et al., 2021; Posner et al., 2005; Posner et al., 2009).

In naturalistic scenarios, display of emotions is highly context-dependent and reveals a high variability over the course of time (between, but also within individual sentences). Therefore, stimuli were rated not only in a post-presentation rating but also continuously during the listening to capture the variabilities of their valence and arousal. To provide a comprehensive description for each stimulus, we also include post-presentation ratings regarding a discrete emotion classification (i.e., basic emotions as well as various discrete emotions with associated intensity evaluation) and potential moderators (e.g., familiarity). We validate the database regarding the three criteria of differentiation, sufficient salience/strength, and generalizability across participants, and propose Monte Carlo simulation (MCS)-based subtractive comparisons to verify an optimal stimulus selection.

## Methods

### Stimuli

Initial stimuli were selected with the criteria that (i) there was no music in the audio sequence, (ii) the language was free of accent, and (iii) emotionally negative and positive audio sequences had high emotional intensity but were not ethically critical in content. Humorous and amusing audio sequences were chosen to elicit positive emotions, since other positive emotions such as love or passion might be more tailored to personality and individual experiences and require a longer time course of exposure. A preselection of 60 audio sequences fulfilling the above-stated criteria (no background music, free of accent, and high in emotional intensity) was obtained via polls published in private social media groups of the students and employees of the university department. With this approach we intended to obtain a relatively diverse initial set. Nineteen of the audio sequences were associated with negative emotions, 25 associated with positive emotions, and 16 associated with neutral emotions. Two independent raters (27 [first author] and 24 years old, both female and German natives) rated the preselection using a scale from 0 (bad) to 10 (good) and provided justification for their rating. Audio sequences with an average rating below 6 were excluded. Reasons for exclusions were, among others, too short duration of the audio sequences and insufficient audio quality or speech intelligibility. The interclass correlation between the raters examined using the Spearman rank correlation coefficient was ${r}_{s}$= 0.92 with a full agreement between the two raters in 48.3% of the cases. The final stimulus pool presented in the validation study comprised a total of 37 audio sequences covering in total duration 92 minutes with 11 associated with negative, 11 with positive, and 15 with neutral emotions. The average of the initial suitability rating across these audio sequences was 7.37 (*SD* = 1.0, range: 6 to 10). The audio sequences were on average 148.8 seconds long (*SD* = 64.12, range: 59 to 393 seconds) and normalized to an average amplitude of −20 dBFS (decibels relative to full scale) using the RMS (root mean square) measure as implemented in pydub (version 0.25.1; Robert, 2011). Detailed information about each audio sequences is provided in Table 1 in the Supplementary Material. All audio sequences are made available on request for scientific use in the OSF project repository GAUDIE https://osf.io/xyr6j/.

### Participants

Twenty-six healthy volunteers (mean age = 24.69 years, *SD* = 3.41, range: 19 to 33 years, 19 females, 7 males) participated in the validation study at the University of Oldenburg, Germany. Only participants reporting German as their mother tongue, no neurological diseases or psychiatric disorders, as well as no consummation of centrally effective medication or drugs, were invited. Regarding their educational level, 50% of the participants at least graduated from high school (here the German Abitur), while 38.5% reported a bachelor’s and 11.5% a master’s degree as their highest educational level.

Previous studies reported effects of personality trait differences on the neuronal responses associated with emotion processing (e.g., emotional word recognition, Ku et al., 2020; and emotional image processing; Kehoe et al., 2012) and on the relationship between valence and arousal ratings (Kuppens et al., 2017). Kanske and Kotz (2012) described the influence of depression and anxiety on valence and arousal ratings of auditory affective words in a non-clinical sample. The authors emphasized the importance of including such psychological characteristics of the rating samples when validating databases to enable subsequent studies to evaluate the match of their sample to the original rating sample (Kanske & Kotz, 2012). Therefore, we recorded and report personality traits, subjective well-being, and anxiety to provide a comprehensive description of the sample characteristics. Participants completed questionnaires to assess their personality via the short version of the German Big Five Inventory (BFI-K; Rammstedt & John, 2005), subjective well-being via the WHO-5 (Psychiatric Research Unit & WHO Collaborating Centre in Mental Health, 1998), and Satisfaction With Life Scale (SWLS; Janke & Glöckner-Rist, 2012), depression via a two-item inventory (Whooley et al., 1997), and anxiety via the German State-Trait Anxiety Inventory (STAI; Laux et al., 1981). Results of the personality scores as well as scores regarding the average subjective well-being and self-reported anxiety indicated a healthy sample and are reported in detail in the Supplementary Material (Supplementary Figure 1, Supplementary Figure 2, and Table 2). The internal consistencies using Cronbach's α of the used scales are reported in Table 3 in the Supplementary Material. Participants gave their written informed consent according to the recommendations of the Declaration of Helsinki before participation and received monetary compensation. The study protocol was approved by the ethics committee of the University of Oldenburg, Germany (Drs. EK/2018/070).

### Procedure

Participants listened to only half of the audio sequences to ensure that the session did not exceed two hours. In each session, the number of stimuli was balanced across conditions and stimulus order was randomized. Each participant provided a continuous rating during and a post-presentation rating after the presentation of each audio sequence. We instructed participants to evaluate what listening to the audio sequences triggered in them personally, rather than to rate the displayed feelings of protagonists in the audio sequence (see Supplementary Material for the written German instructions and English translation).

For the continuous rating—that is, when listening to the audio sequence—an adapted version of the Self-Assessment Manikin (SAM; Bradley & Lang, 1994) subscale of valence and arousal with a slider below the pictorial rating scale was used (see Supplementary Figure 3). Each audio sequence of the individual selection was presented twice to the participants. In the first presentation, valence was rated, and in the second, arousal. We chose this order because we assumed that repetition might affect the evaluation of valence more strongly than the evaluation of arousal. Moreover, at least one other sequence was presented between the two presentations. The slider position was sampled at 1 Hz, mapped on a scale between 0 and 100, and was placed at stimulus start in the middle of the scale.

The post-presentation ratings of the audio sequences which were taken after the presentation included multiple rating scales. After the first presentation of the audio sequence, participants selected the prevailing emotion they experienced during the presentation on an adapted version of the Geneva Emotion Wheel (GEW; version 3.0, Sacharin et al. 2012) with an intensity rating on a 6-point Likert scale (see Supplementary Figure 3). We removed the option to choose neutral, because we wanted to obtain the direction of a neutral audio sequence with a forced choice. A neutral evaluation of the experienced emotion could still be expressed via the intensity of the chosen emotion. Next, after the GEW rating, they had to select the prevailing emotion out of the six basic emotions (joy, anger, disgust, fear, sadness, and surprise, Ekman, 1992; Izard, 2007).

Many neurophysiological studies investigating emotional processing (e.g., Lithari et al., 2010; Posner et al., 2009; Rozenkrants & Polich, 2008) and database validation (Belin et al., 2008; Lima et al., 2013) focused only on two SAM dimensions—valence and arousal. Bakker et al. (2014), however, strongly advocate in their review the inclusion of the third SAM dimension, dominance, to comprehensively evaluate the emotional processing. We decided, thus, to include dominance to explore differentiation of the stimulus types, but only as a short post-presentation scale to save time. Hence, after the second presentation, participants were asked to rate the experienced dominance of the previously presented audio sequence via the SAM rating, and how familiar they were with the audio sequence, using a slider ranging from 0 to 100 with the starting point at 50. A major rationale for including dominance only as post-presentation rating was that an assessment of one’s perceived control and dominance triggered by the stimuli might be difficult and potentially biased since it is strongly affected by the experimental-based inescapable exposure. Previous studies using non-speech auditory stimuli, and focusing less on the sensations induced in the raters, reported that the dominance ratings mirrored the valence dimension (Bradley & Lang, 2007; Yang et al., 2018). Because of the differences in task instruction and stimulus type, we here expect a similar evaluation to the arousal dimension, with dominance representing the extent to which a stimulus engages and captures the listener's attention.

### Statistical analysis

#### Descriptive statistical analysis

In the first part, the stimulus database was analysed descriptively using (a) the SAM ratings with the subscales valence and arousal, as well as the post-presentation subscale dominance, and (b) the additional post-presentation ratings (familiarity, basic emotions, GEW emotions, and strength of the GEW emotions).

##### SAM ratings

For the continuous valence and arousal ratings, we visualized each audio sequence as a time series by calculating the bootstrapped mean and its 95% confidence interval (CI) per time point across participants. In the bootstrapped time series, we observe an orientation phase at the beginning of each audio sequence. In this orientation and response adaption phase, participants first learned about the context and protagonists of the audio sequence and moved the slider, initially positioned at the middle of the scale, little in all three conditions. After approximately 30 seconds, there is a shift from the average starting point of the slider towards lower valence ratings in the negative condition and higher ratings in the positive condition. In further analyses, we, therefore, removed this time interval of orientation and response adaption in all three conditions. Next, we computed the grand average and its 95% CI of the mean and standard deviation for each audio sequence by first averaging over the time series within each participant and then bootstrapping with 5000 iterations across participants. The latter step was also applied to the dominance ratings, allowing us to obtain the bootstrapped mean and its 95% CI over participants. The grand average over audio sequences of the same condition was calculated as a reference for within-class similarity. In the next descriptive analysis using the SAM scales, we introduce a clustering approach to further examine separation and discrimination among audio sequences and conditions using fuzzy c-means (Bezdek et al., 1984; Nayak et al., 2015). We used a predefined number of three clusters representing the three conditions and the Python fuzzy c-means toolbox (version 1.6.4; Dias, 2019). The fuzzy c-means algorithm allows for a fuzzy partitioning into clusters. This algorithm assigns a probability of belonging to a certain cluster to each audio sequence. This probability of cluster affiliation can be used as a score to assess how representative a data point is of a cluster. To determine the cluster affiliation, those with the highest probability are chosen. The matching between cluster and condition was determined by the majority of audio sequences belonging to one experimental condition within the respective cluster. From the obtained probability scores of the ratings per audio sequence, we calculated its mean and 95% CI for each cluster affiliation via bootstrapping with 5000 iterations. With good separation, the lower bootstrapped CI of the probability associated with the true cluster affiliation (i.e., the condition of the respective audio sequence) should not overlap with the CI of the probabilities for the other clusters (mainly the neighbouring neutral condition).

In a final analysis of the SAM ratings, we were interested in increasing the differences between ratings of the emotional audio sequences and those of the neighbouring condition (i.e., the neutral condition), by choosing only particularly distinct audio sequences. These are audio sequences revealing a large difference from those of the neighbouring neutral condition and small difference from audio sequences of the same emotional condition. To evaluate how well each emotional audio sequence was discriminated from neutral audio sequences in their SAM ratings, we performed repeated pairwise subtractive comparisons via MCS (Ernst, 2004) per subscale. In the first step, all possible combination pairs of emotional and neutral audio sequences were generated (total combination pairs of *N* = 330 with ${n}_{negative-neutral}=165$ and ${n}_{positive-neutral}=165$). For each combination pair, we created a vector of 5000 randomly selected ratings of the emotional audio sequence and subtracted it from a vector of 5000 randomly selected ratings of the neutral audio sequence, thereby ignoring the within-subject factor. Based on the hypothetically assumed relationship of both audio sequences, we identified combination pairs revealing less distinct separation. For this purpose, we calculate the percentage of occasions per combination pair in which the hypothetically assumed relationship of both audio sequences was violated. For the valence ratings, we assumed increased values from negative to neutral to positive. For the arousal and dominance ratings, we assumed higher values for emotional (negative and positive) compared to neutral audio sequences. We visualized those pairs with a probability of misclassification above 20%. In application cases where a particularly distinct pool of audio sequences per condition is required, this analysis allows justification to select a subset of audio sequences.

##### Additional post-presentation ratings

In the second part of the descriptive analysis, the database was analysed regarding the additional post-presentation rating scales, comprising familiarity, basic emotions, GEW emotions, and strength of the GEW emotions.

Frequencies of the GEW and basic emotions ratings were normalized between 0 and 1, and visualized using radar charts per condition and each audio sequence. We computed four additional scores from the GEW and basic emotions ratings describing whether the participant rated the audio sequence negative or positive. Therefore, we first classified whether the chosen emotion was associated positively or negatively. Regarding the GEW ratings, the emotions interest, joy, pleasure, satisfaction, amusement, compassion, pride, and relief were classified as positive, whereas fear, disgust, hatred, anger, disappointment, sadness, contempt, shame, guilt, and regret were classified as negative. Regarding the basic emotions, joy and surprise were classified as positive and sadness, fear, disgust, and anger as negative. This resulted in four additional scores (i.e., negative basic emotion, positive basic emotion, negative GEW emotion, and positive GEW emotion). In the next step, only the GEW emotion scores were weighted by the strength of the perceived response using the GEW intensity rating.

#### Inferential statistical analysis

Finally, the three stimulus conditions were statistically compared regarding their SAM ratings and additional post-presentation ratings (i.e., familiarity, basic emotions, negative GEW emotion, positive GEW emotion, GEW strength/intensity) using repeated-measures analyses of variance (rmANOVAs; toolbox pingouin; version 0.5.1; Vallat, 2018) with the factor condition (negative vs. neutral vs. positive). Post hoc comparisons between conditions were performed by computing the difference participant-wise as well as the Bonferroni-corrected CI for each contrast via bootstrapping. Contrasts where the CI does not include zero can be considered as significant (Cumming & Finch, 2005).

## Results

### Descriptive and inferential analysis using the SAM ratings

**Fig. 1:**
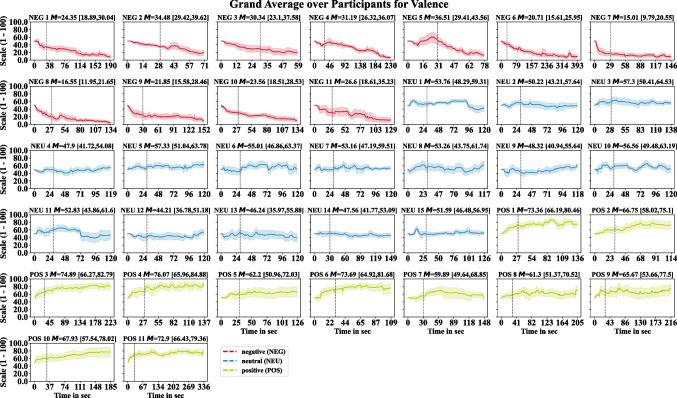
Bootstrapped grand averages for the continuous valence ratings among participants per audio sequence over the time course of listening. Shaded areas represent the bootstrapped 95% confidence interval (CI) of the mean deviation over participants. The mean (M) over the time course with its 95% CI are provided in brackets in the subtitles. The dashed vertical line indicates the initial 30-second adaptation phase

**Fig. 2:**
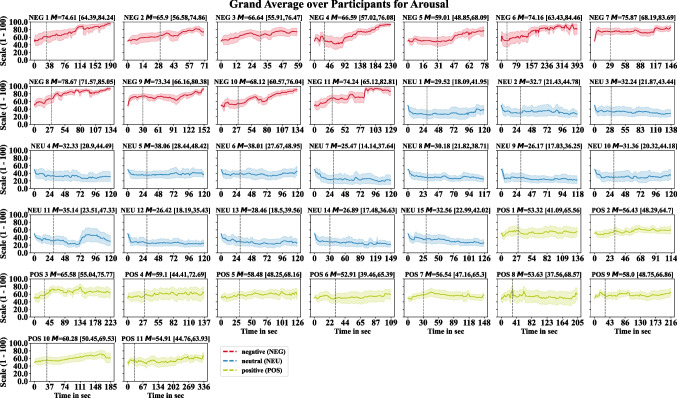
Bootstrapped grand averages for the continuous arousal ratings among participants per audio sequence over the time course of listening. Shaded areas represent the bootstrapped 95% confidence interval (CI) of the mean deviation over participants. The mean (M) over the time course with its 95% CI are provided in brackets in the subtitles. The dashed vertical line indicates the initial 30-second adaptation phase

To investigate the variability of the ratings over the time course of listening, Figs. 1 and 2 illustrate the bootstrapped mean and its 95% CI per time point across participants for each audio sequence for the continuous valence and arousal ratings. After the orientation and response adaption phase, lower valence ratings in the negative condition and higher ratings in the positive condition can be observed. For the continuous arousal ratings, we observed lower values for the neutral audio sequences compared to the negative and positive condition as well as lower arousal values for the positive when compared to the negative condition. Nevertheless, we observe dynamic variation in the temporal course of listening for both continuous ratings, which illustrates the importance to keep the evaluation for dynamic, complex stimuli at the level of individual time points. We also examined whether the position within a session affected the continuous rating. No correlation between ratings and their position was observed for valence (${r}_{p}$= −0.03; $p$= 0.471) and arousal (${r}_{p}$= 0.002; $p$= 0.972).

**Fig. 3:**
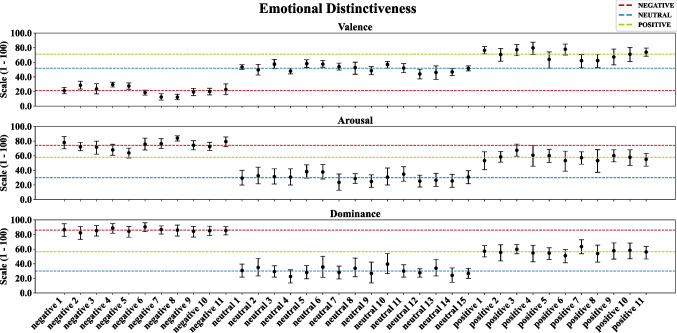
Bootstrapped average valence, arousal, and dominance ratings as well as 95% confidence interval (CI) per audio sequence. Dashed lines represent the mean overall stimuli in each emotion condition

To examine the strength of the induced emotional evaluation, generalizability across participants, and differentiation to audio sequences of the neighbouring conditions,

Figure 3 shows for each stimulus the bootstrapped grand average over time and participants of the continuous valence and arousal ratings, as well as the grand average over participants of the post-presentation dominance ratings, excluding the initial 30-second adaptation phase. Error bars indicate the 95% CI of the grand average. The horizontal dashed lines represent the grand average over all stimuli of each emotion condition.

Generally, the SAM ratings are similar within condition and exhibit good separation between conditions. However, the CIs of the valence ratings for the positive audio sequences 5, 7, and 8 slightly touch the grand average of the neutral emotion condition, suggesting that these stimuli may be rated neutral by some participants. Similarly, we observed for arousal ratings slight overlaps of the CIs of the audio sequences negative 5, positive 3, and positive 4 with the grand average of the opposite emotional condition. Regarding the post-presentation dominance ratings, we observed good separation between all three conditions with highest ratings for the negative audio sequences, followed by positive sequences, and the lowest ratings for neutral audio sequences. Detailed information on the mean and CI per audio sequence and rating is provided in Table 4 in the Supplementary Material. Results of the rmANOVA revealed strong significant differences of the average ratings per condition for all three SAM scales with *F*(2,50) = 160.22, *p* < 0.001, ${\eta }_{p}^{2}$ = 0.87 for the valence, *F*(2,50) = 71.73, *p* < 0.001, ${\eta }_{p}^{2}$ = 0.74 for the arousal, and *F*(2,50) = 134.41, *p* < 0.001, ${\eta }_{p}^{2}$ = 0.84 for the dominance ratings. Bootstrapped mean differences and Bonferroni-corrected 2.5^th^ and 97.5^th^ CIs of the mean per contrast are provided in Table 10 in the Supplementary Material.

**Fig. 4:**
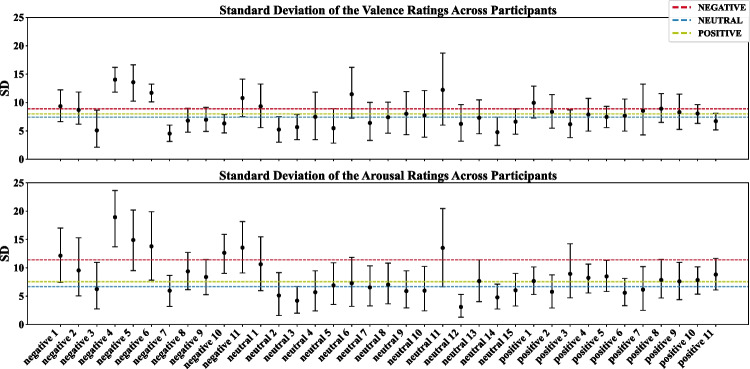
Bootstrapped average standard deviation (SD) of the valence and arousal ratings as well as its 95% confidence interval (CI) per audio sequence. Dashed lines represent the mean over all stimuli in each emotion condition

The consistency of ratings within each audio sequence is indicated by the standard deviation of the continuous ratings over time. Figure 4 provides the bootstrapped average standard deviation and respective 95% CI of the valence and arousal ratings for each stimulus across participants (see also Table 5 in the Supplementary Material). For the valence ratings, we observed comparable variations within the ratings over time across conditions, with some outliers revealing larger variability (negative 4 and 5 as well as neutral 6 and 11), but no statistically significant difference between the conditions, *F*(2,50) = 1.88, *p* = 0.163, ${\eta }_{p}^{2}$ = 0.07 (see Table 10, Supplementary Material). The standard deviation of the arousal ratings is significantly higher for the negative condition than for the neutral and positive condition, indicating that the arousal ratings of the negative stimuli are less consistent over the time course of the audio sequence, with *F*(2,50) = 15.20, *p* < 0.001, ${\eta }_{p}^{2}$ = 0.38 (see Table 10, Supplementary Material).

**Fig. 5:**
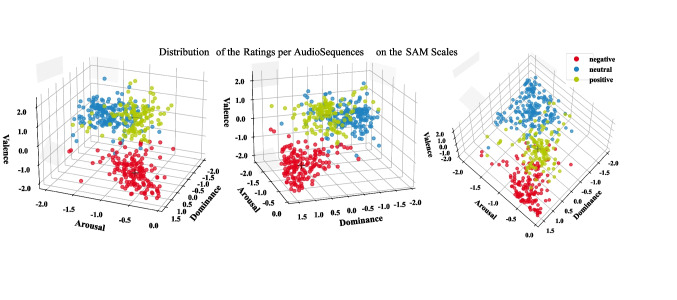
Distribution of the clustered z-score normalized data on the three SAM scales of valence, arousal, and dominance. Each data point represents a clustered audio sequence rating of one participant. Colours represent the cluster label

Next, we investigated the separability and clustering of the stimuli in the full SAM space with its three dimensions instead of analysing each dimension separately. To quantify the separation of the audio sequences of the three conditions, we performed an unsupervised fuzzy c-means clustering with three clusters and the SAM ratings as feature set. The cluster assignment had a 93.02% correspondence with the a priori defined conditions, indicating that the different stimuli form the expected clusters on the SAM ratings and are well separated between clusters. The matching between cluster and condition was determined by the majority of audio sequences belonging to one experimental condition within the respective cluster. Figure 5 illustrates the distribution of the clustered data in a three-dimensional space where each axis represents a SAM scale, and each point a stimulus rating from one participant. The data clearly fall into three clusters representing the three conditions. The red point cloud representing the negative condition appears to be separated by the largest margin from the other clusters. The positive (green) and neutral (blue) clusters are less separated.

We further analysed the separability of stimuli that were misclassified using the probability of cluster affiliation per cluster. Fuzzy c-means allows the computation of the probability of the assignment to each of the three clusters. We calculated the bootstrapped average probabilities and CIs across participants per cluster affiliation of audio sequences which were at least once (i.e., for at least one participant) wrongly assigned based on the fuzzy c-means clustering. For each audio sequence, we marked the lower CI of the average probability of the true cluster affiliation across participants as a threshold (see Supplementary Figure 4). In case the CI of the average probability for belonging to one of the other two clusters overlaps with or touches this threshold, the audio sequence is less clearly differentiated from neighbouring clusters. Using this logic, only the positive 8 audio sequence is identified as less separable from one other cluster (i.e., the neutral condition) in the SAM rating space (probability for the neutral cluster: *M* = 0.29 [0.17; 0.42]; probability for the negative cluster: *M* = 0.56 [0.40; 0.72]; see Supplementary Figure 4 and Table 6 in the Supplementary Material for further values of the bootstrapped means and CIs). This indicates that even when the stimulus ratings of single participants lead to wrong emotion classifications, the stimuli are well separated at the level of the average SAM ratings in the participant population.

**Fig. 6:**
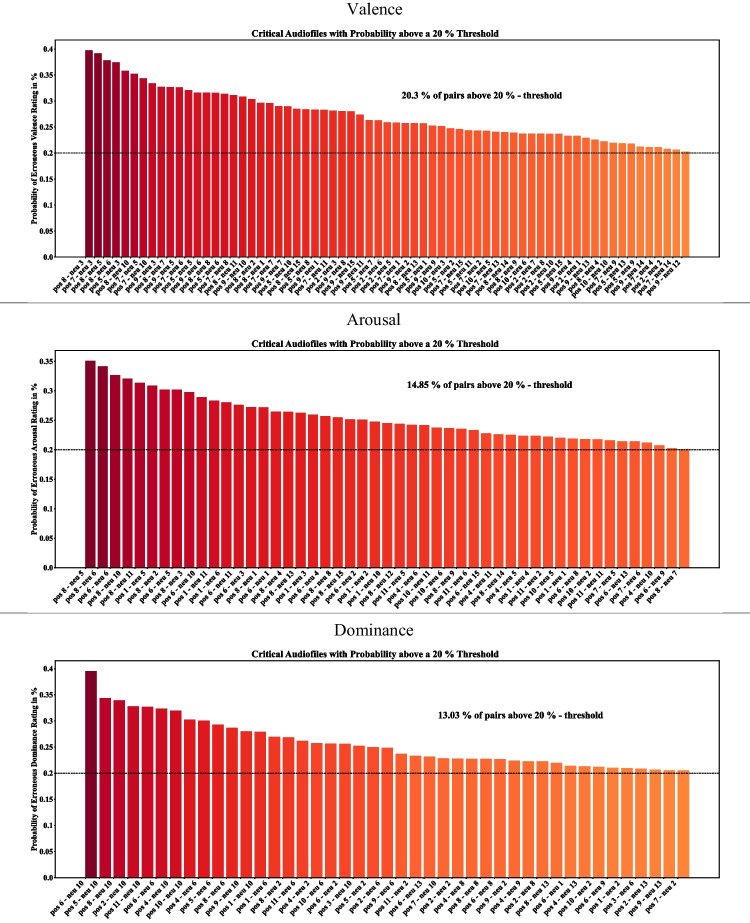
Repeated pairwise subtractive comparisons via a Monte Carlo simulation (MCS) with 5000 iterations. The depicted stimulus pairs have a probability of erroneous evaluation above 20%. Dashed lines represent the predefined threshold of 0.2 indicating 20% probability of an erroneous rating relationship between the combination pair. Pos = positive, neu = neutral, neg = negative

Finally, we investigated which audio sequences could be removed from the stimulus set, if only a subset of stimuli is required in an experiment. In our approach, we simulate the discriminability of stimulus pairs on the SAM ratings using a Monte Carlo Simulation (MCS, see Methods) to identify audio sequence pairs particularly suitable for removal due to lower discriminability. In Fig. 6, we visualize those stimulus pairs which revealed erroneous rating relationships with a probability of at least 20% in the simulation; separately for each SAM scale. This rather complex listing of misclassification rates among stimulus pairs can be broken down by analysing which stimulus pairs were misclassified with a probability above 20%. For the valence ratings (Fig. 6, upper row), 20.3% of all combination pairs revealed a probability of misclassification above the threshold of 20%. The top four audio sequences with the highest probabilities of misclassification were positive 7, positive 8, neutral 3, and neutral 5 and were, therefore, identified as suitable candidates for removal if the valence scale is of importance. For the arousal ratings, we observed above-threshold misclassifications in 14.85% of the combination pairs, with positive 8, neutral 6, neutral 5, and positive 6 being the top four with the most repetitions and highest probabilities (Fig. 6, middle row). For the dominance ratings, misclassification above the 20% threshold occurred in 13.03% of the combination pairs. The top four audio sequences with the highest probabilities of misclassification were neutral 10, neutral 6, neutral 2, and positive 6, indicating them as suitable candidates for removal (Fig. 6, lower row).

### Descriptive and inferential analysis using additional post-presentation ratings

**Fig. 7:**
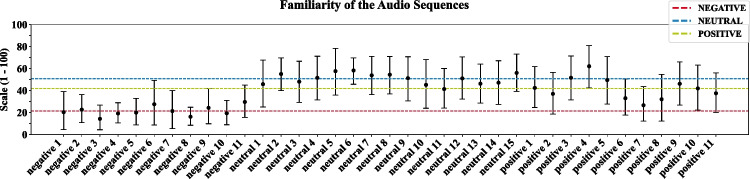
Bootstrapped averaged familiarity and its 95% confidence interval (CI) per audio sequence. Dashed lines represent the grand average within the respective condition

Since familiarity of a stimulus might affect other ratings, we analysed how familiar the participants were with the audio sequences. On average, the highest ratings were assigned to stimuli of the neutral condition, followed by stimuli of the positive emotion condition. The negative emotion condition received on average the lowest familiarity ratings (see Fig. 7 and Supplementary Material, Table 7) and differed significantly from average ratings of neutral and positive stimuli, *F*(2,50) = 13.89, *p* < 0.001, ${\eta }_{p}^{2}$ = 0.36 (bootstrapped contrasts with negative–neutral: *M* = −28.87 [−39.62; −18.2] and negative–positive: *M* = −21.01 [−33.94; −8.38]; but positive–neutral (n.s.): *M* = −7.85 [−23.56; 8.09]; see Table 10 in the Supplementary Material). To examine whether familiarity of the audio sequence influences its SAM ratings, we correlated the SAM ratings with familiarity per condition (see Table 8 in the Supplementary Material). None of the correlations was significant. We observed a non-significant trend towards a positive correlation between familiarity and valence ratings only for the positive stimuli.

**Fig. 8:**
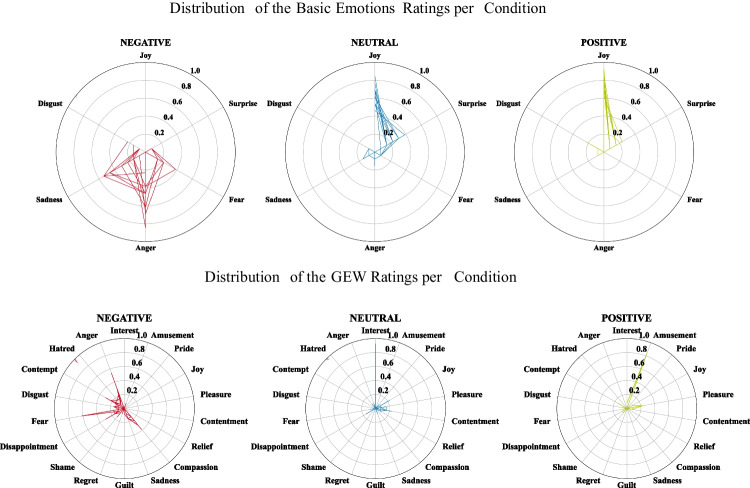
Frequencies of the normalized basic and GEW emotions ratings per condition with a scale ranging from 0 to 1. Each layer represents the ratings of one stimulus

We now turn to the analysis of the discrete emotion ratings, the GEW, and the basic emotions classes. Figure 8 shows the distribution and frequencies of chosen emotions for the basic emotions (upper) and GEW ratings (lower), separately for the three stimulus types. We observe a wider distribution of basic emotions chosen for the negative stimuli which distributes over fear, anger, sadness, and disgust. Similarly, the GEW ratings scatter over a wide range of predominantly negative emotions including anger, contempt, disgust, fear, disappointment, shame, and sadness, but also compassion. For the positive and neutral stimuli, we observe less variations in the chosen emotions. Among the basic emotions, mainly joy and surprise were selected, with little differentiation between stimulus types. However, positive and neutral stimuli separated on the GEW scale. There, amusement was most often chosen for the positive and interest for the neutral condition. The distributions and frequencies per stimulus are provided in the Supplementary Material, Supplementary Figure 5 to Supplementary Figure 10.

**Fig. 9:**
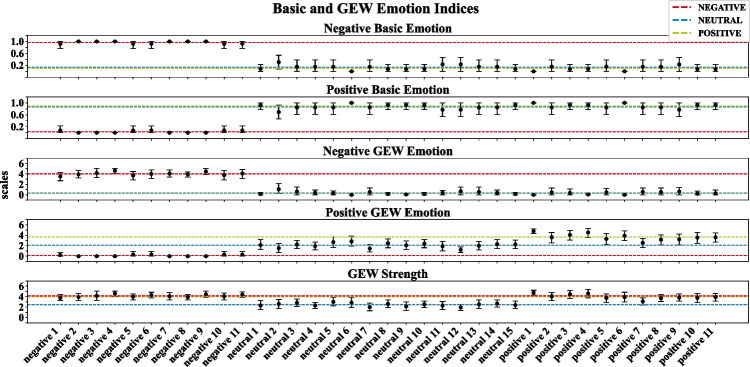
Bootstrapped average of the basic and GEW emotions ratings with its 95% confidence intervals (CI) per audio sequence. Dashed lines represent the grand average within the stimulus classes

Figure 9 shows the basic emotions and the GEW ratings averaged across participants, separately for each stimulus. In order to reduce the complexity, we combined the ratings separately for the negative and positive emotions in each discrete emotion system. The negative and positive basic emotions ratings clearly differentiated the negative from the positive and neutral stimuli, with *F*(2,50) = 170.50, *p* < 0.001, ${\eta }_{p}^{2}$ = 0.87. However, there was no significant difference between the average ratings of the positive and neutral stimuli, with a bootstrapped mean of *M* = 0.03 and Bonferroni-corrected CIs including the zero; [−0.12; 0.16]. We observe a similar strong differentiation of negative from positive and neutral stimuli in the GEW ratings, with *F*(2,50) = 231.70, *p* < 0.001, ${\eta }_{p}^{2}$ = 0.9 for the negative and *F*(2,50) = 70.21, *p* < 0.001, ${\eta }_{p}^{2}$ = 0.74 for the positive GEW ratings. We observed a significant stepwise difference only for the positive GEW score, with the lowest values in the negative condition, followed by the neutral condition, and the highest values in the positive condition (bootstrapped contrasts with negative–neutral: *M* = −1.95 [−2.52; −1.39]; positive–neutral: *M* = 1.6 [0.66; 2.38]; negative–positive: *M* = −3.55 [−4.11; −2.9]; see Table 10 in the Supplementary Material). The results suggest that the GEW ratings allowed for somewhat better differentiation between the neutral and positive condition compared with the basic emotions ratings. The GEW strength rating clearly differs between neutral and emotional stimuli, *F*(2,50) = 38.60, *p* < 0.001, ${\eta }_{p}^{2}$ = 0.61, with lower ratings for the neutral than the negative and positive stimuli. However, no difference was found between positive and negative stimuli (bootstrapped difference negative–positive: *M* = 0.13 [−0.26; 0.54]; see Table 10 in the Supplementary Material). Numerical values of the bootstrapped means and CIs per audio sequence for the basic emotions and GEW ratings are provided in Table 9 in the Supplementary Material.

## Discussion

We here developed, validated, and characterized a novel German database of naturalistic and continuously rated auditory stimuli comprising emotionally neutral, positive, and negative speech sequences. The database aims to fill a gap in the existing databases of emotion-inducing stimuli. We demonstrate that the stimuli fulfil the criteria of a sufficient level of salience, intensity, and intrusiveness and are well separated on the valence-arousal-dominance system of emotions.

The analyses chosen in this work aim to provide (a) multiple annotations per audio sequence which can also be used for further validations or for choosing a specific selection of audio sequences and (b) an estimation of robustness across evaluation methods and participants. The rich annotations per audio sequence is a particular advantage of the database, with both post-presentation ratings and continuous annotations for valence and arousal. These annotations allow, among others, further correlation analyses with peripheral and neurophysiological reactions evoked by the processing of the audio sequences.

The participants’ post-presentation basic emotions classifications did not differentiate between neutral and positive audio sequences; only negative stimuli were categorized with different emotions. Consequently, a categorical assessment based on the basic emotions may not be sufficient to distinguish between emotional positive and neutral audio sequences. However, the GEW ratings, featuring a more fine-grained differentiation of emotions categories and a continuous intensity scale, distinguished between positive and neutral audio sequences, especially through the intensity ratings. Interestingly, we observed a difference in the familiarity post-presentation ratings for the negative compared to the positive and neutral conditions. The higher familiarity ratings for the neutral audio sequences potentially indicate that participants are more familiar with the general type of audio sequence—that is, weather forecasts—than with the specific sequence. Importantly, familiarity did not affect the SAM ratings significantly. One limitation of the neutral audio sequence is the higher information density conveyed within the sequence. Although the conveyed information is potentially irrelevant and therefore less salient, the processing complexity increases.

A further limitation is the young participant sample with a high proportion of female university students recruited for validation. Since the validation using multiple scales and continuous ratings was quite time-intensive, only half of the database was rated by a participant. Although our results revealed rather good consistency across participants in the differentiation between stimulus types, ratings might still have been influenced by age or potential gender differences. However, the participant sample recruited for validation fulfilled specific inclusion criteria (e.g., young age below 35 years and no diagnosed psychological disorders) to approximate samples often used in neurophysiological studies. It is therefore important to note that the results of the validation may not be representative of the whole population but rather limited to a subsample of the population matching the characteristics of the validation sample. The characterization of the stimuli can be extended in the future. For example, the relationship between the ratings and auditory features such as pitch, timbre, and loudness as well as speech-related features (e.g., spectral and temporal modulation features; Santoro et al., 2014; Boos et al., 2021) of the sequence could be a valuable extension. Moreover, information about the frequencies of the words used in the audio sequences as well as speed of speech could further widen the field of research in which the GAUDIE database is applicable.

## Conclusion

We hereby provide a validated and richly annotated naturalistic affective German speech stimulus database named GAUDIE which can be used to induce positive, neutral, and negative emotion. The multiple continuous and discrete ratings can be further used to investigate influencing auditory features as well as the relationship between behavioural emotional evaluations and evoked (neuro-)physiological reaction when listening to the audio sequence. The suggested audio sequences are accessible on request for further research (https://osf.io/xyr6j/) and fulfil criteria of differentiation, sufficient salience/strength, and generalizability. We hope that the stimulus set is useful for the research community and want to encourage researchers to add further annotations, which can be linked with the audio sequences.

## Data Availability

The datasets generated and analysed during the current study are made available on request via the OSF project repository GAUDIE https://osf.io/xyr6j/.

## Code availability

The GitHub repository with the code used to analyse the current study is available in the OSF project repository GAUDIE https://osf.io/xyr6j/.
